# Autoimmune Encephalitis in Tunisia: Report of a Pediatric Cohort

**DOI:** 10.1155/2021/6666117

**Published:** 2021-05-10

**Authors:** Bissene Douma, Thouraya Ben Younes, Hanene Benrhouma, Zouhour Miladi, Imen Zamali, Aida Rouissi, Hedia Klaa, Ichraf Kraoua, Melika Ben Ahmed, Ilhem Ben Youssef Turki

**Affiliations:** ^1^Research Laboratory LR18SP04 and Department of Child and Adolescent Neurology, National Institute Mongi Ben Hmida of Neurology, Tunis, Tunisia; ^2^Université de Tunis El Manar, Faculté de Médecine de Tunis, 1007 Tunis, Tunisia; ^3^Department of Clinical Immunology, Pasteur Institute, Tunis, Tunisia

## Abstract

**Background:**

Autoimmune encephalitis (AE) is a rapidly progressive encephalopathy caused by antibodies targeting neurons in the central nervous system generating specific immune responses. It is increasingly recognized in children.

**Objective:**

To describe clinical, neuroimaging, and laboratory features, treatment, and outcome in a cohort of Tunisian children with AE.

**Methods:**

We conducted a retrospective review of the medical records of all children attending the Department of Child and Adolescent Neurology (Tunis) with autoimmune encephalitis between 2004 and 2020. Clinical, neuroimaging, laboratory features, therapeutic data, and outcome were analyzed.

**Results:**

Nineteen children were included in the study (12 girls and 7 boys). The median age at diagnosis was 7.68 years (range: 10 months-13 years). The most frequent manifestations were seizures and behavioral disorders. Eleven cases were diagnosed with anti-NMDA receptor encephalitis, 4 cases with anti-Ma2 encephalitis, 3 cases with anti-GAD encephalitis, and 1 case with anti-SOX1 encephalitis. Brain MRI showed increased T2 and fluid-attenuated inversion recovery (FLAIR) signal of the temporal lobe in 5 patients. Eighteen patients showed improvement following first-line immunotherapy (high-dose corticosteroids, intravenous immunoglobulin). One patient with anti-GAD encephalitis died despite escalating immunotherapy.

**Conclusion:**

Diagnosis of autoimmune encephalitis is challenging in children, because of misleading presentations. An early and accurate diagnosis is important to enable proper therapeutic interventions.

## 1. Introduction

Autoimmune encephalitis (AE) represents one of the most common causes of noninfectious encephalitis. In the past 10 years, an increasing number of AE cases have been reported [1]. The clinical presentation of AE in childhood is subacute with a varied constellation of symptoms [2–4]. Brain magnetic resonance imaging (MRI) may demonstrate abnormalities that provide clues for diagnosis [2, 5]. The identification of specific autoantibodies was a major advance achieved in neurology. Seronegative AE had been reported [4]. The outcome of AE in childhood is generally good [2]. In Tunisia, there was no published series of pediatric AE.

The aim of the present study was to investigate clinical features, biological and radiological aspects, management, and outcome of Tunisian children with AE.

## 2. Patients and Methods

We conducted a retrospective and descriptive study over 17 years (between 2004 and 2020) in the Department of Child and Adolescent Neurology at the National Institute Mongi Ben Hmida of Neurology (Tunis, Tunisia).

Patients with acute or subacute neurological disorders were considered eligible for this study if they fulfilled the consensus diagnostic criteria for autoimmune encephalitis in adults [1] and revised based on the newly proposed diagnostic criteria in pediatric patients [6].

The exclusion criteria included patients with evidence of infectious encephalitis, for example, viral, bacterial, Mycobacterium tuberculosis, or fungal.

Antibodies were detected using indirect immunofluorescence by commercialized slides with a mosaic of biochips (Euroimmun®), each one containing transfected cells expressing the receptors of a different neuronal surface antigen: NMDA, AMPA, GABAB, CASPR2, and LGI1. Antibodies against Cv2, Ma2, Ri, Yo, Hu, recoverin, titin, SOX1, and amphiphysin were tested by the commercial immunoblot kit EUROLINE Paraneoplastic Neurological Syndromes 12 Ag (DL 1111-1601-4 G; Euroimmun, Lübeck, Germany) following the manufacturers' instructions at serum dilution 1/100. Antibodies against GAD65 were detected using a commercialized enzyme-linked immunosorbent assay from Euroimmun®.

Medical records of patients with AE were retrospectively reviewed. Demographic characteristics, clinical data, biological findings, characteristics of brain magnetic resonance imaging (MRI), and the data about therapeutic management and outcome were collected.

First-line immunotherapy included intravenous (IV) methylprednisolone or intravenous immunoglobulins (IVIG), or a combination of these. Rituximab or azathioprine was defined as second-line immunotherapy. All patients were followed for at least 3 months (in the range of 3 months-9.5 years). Good outcome was defined as no sequela, and poor outcome as having any sequela.

A descriptive analysis was performed using SPSS software. Data are expressed as means.

## 3. Results

Nineteen children were included in our study. The male-female ratio was 0.58 (12 girls and 7 boys). Based on the proposed diagnostic criteria for autoimmune encephalitis [1, 6], all of the patients met a definite diagnosis of autoimmune encephalitis.

Antibodies were detected against NMDAR in 11 cases, against Ma2 in 4 cases, against GAD65 in 3 cases, and against SOX1 in one case.

The median age at diagnosis was 7.68 years (range: 10 months-13 years). There was a personnel medical history of neurofibromatosis type 1 (NF1) in one case with anti-NMDAR encephalitis, epileptic encephalopathy in 1 case with anti-Ma2 encephalitis, and febrile seizure in one case with anti-GAD65 encephalitis.

The majority of patients had subacute onset of symptoms. Eight patients presented with prodromal symptoms, including fever and headache. Two cases with anti-NMDAR encephalitis had previous herpes simplex encephalitis diagnosed by polymerase chain reaction (PCR).

The most preponderant clinical manifestations were seizures, observed in 18 cases, and behavioral disturbances, noticed in all cases. Seizures were focal in 11 cases and generalized in 7 cases.

On examination, a decreased level of consciousness was observed in 11 cases and cognitive dysfunction in 12 cases. Speech disturbances were noted in 12 cases. Movement disorders were objectified in 8 cases with anti-NMADR encephalitis, 1 case with anti-GAD65 encephalitis, and 1 case with anti-SOX1 encephalitis. There were 8 cases of orofacial dyskinesia, 4 cases of dystonia, 2 cases of tremor, 1 case of myoclonia, and 1 case of chorea. Autonomic dysfunction, including dysrhythmia, alternating bradycardia/tachycardia, and hypotension/hypertension, was noticed in one case with anti-GAD65 encephalitis. Hemiparesis was noticed in 2 cases, facial paralysis in 1 case, and opercular syndrome in 1 case.

**Figure 1:**
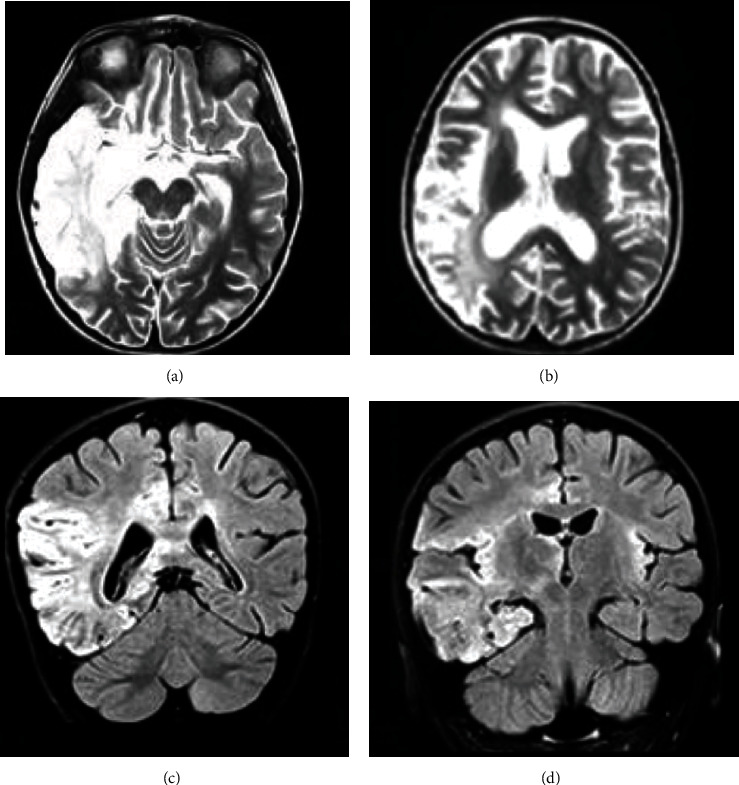
Brain magnetic resonance imaging from a patient with post-HSV-1 autoimmune encephalitis and NMDAR antibodies showed corticosubcortical hyperintensity in the right temporoparietal lobe on T2 weighted (a, b) and fluid attenuation recovery (FLAIR) sequences (c, d).

Brain MRI showed increased signal on fluid-attenuated inversion recovery (FLAIR) and T2-weighted images in 9 patients. Involvement of the temporal lobe was noticed in 5 patients (Figure 1).

Cerebrospinal fluid (CSF) analysis was performed in all patients. Mild hyperproteinorrhachia (maximum 0.84 g/l) was noticed in 2 cases and pleocytosis (maximum 64 lymphocytes/mm^3^) in 7 cases.

The electroencephalogram (EEG) showed abnormalities in 15 cases. There were temporal spike waves in 6 cases, generalized spike waves in 4 cases, and slowing background rhythm in 6 cases.

Oncological assessments were performed in our patients. Tumor markers (alpha-fetoprotein, carcinoembryonic antigen, cancer antigen 19.9, cancer antigen 15.3, and cancer antigen 125), ultrasonography, and thoraco-abdomino-pelvic computed tomography provided no evidence of tumors.

From a therapeutic standpoint, 18 patients received a methylprednisolone pulse (30 mg/kg/day for 5 days) and intravenous immunoglobulin (IVIG) (0.4 g/kg/day × 5 days) and 1 patient was treated with methylprednisone alone. A second-line treatment with rituximab at the dose of 375 mg/m^2^/week was indicated in one case with anti-GAD65 encephalitis. This patient was managed in the intensive care unit because of uncontrollable autonomic imbalance and refractory focal motor seizures. Azathioprine was prescribed in 7 cases.

The mean follow-up period was 3.5 years (range: 3 months-9.5 years). Nine patients achieved good outcomes, while 10 patients had poor outcomes. One child with anti-GAD65 encephalitis deceased six months after onset because of severe dysautonomia.

Detailed clinical, biological, neuroimaging, and therapeutic data and outcome are presented in Table 1.

## 4. Discussion

Our study provides clinical, biological, and radiological characteristics, management, and outcome of AE in a Tunisian pediatric cohort over 16 years.

AE is a rapidly expanding group of diseases with a description of a new subtype appearing every 10 months over the past 10 years [7, 8]. These conditions are characterized by the presence of autoantibodies in serum and/or CSF that are specific and can be used as diagnostic biomarkers. It can be triggered by tumors and infections, or it may be cryptogenic. In our series, herpes simplex encephalitis antedates the development of anti-NMDAR encephalitis in 2 cases.

AE has a wide variety of clinical manifestations including seizures, movement disorders, autonomic disturbances, and behavioral and psychiatric symptoms [3]. In approximately 50% of patients, a prodromal phase is evident days to weeks before disease onset. It may consist of fever, headache, and/or upper respiratory illness [2]. In our series, 8 patients had a prodromal phase. This prodromal phase is followed by psychiatric (hallucinations, paranoia, insomnia, and agitation) and neurological symptoms (seizures, speech impairment, ataxia, and movement disorders). Seizures are the most common symptom, present in up to 80% of patients, and different types of seizures may be observed [2, 3]. In our series, 18 cases had seizures. There were focal seizures in most of our patients. Hyperkinetic movements are frequently noted in pediatric AE. Orofacial dyskinesias, noticed in 8 cases in our series, are commonly seen [7].

Evaluation of a child with suspected autoimmune encephalitis should include both serum and CSF analysis to detect the presence of pathogenic autoantibodies [9]. In NMDAR encephalitis, antibody testing from the CSF has been shown to be more sensitive and specific than serum [10]. In our cohort, 5 cases had anti-NMDAR in the CSF and not in the serum.

Anti-NMDAR encephalitis is the most frequent pediatric AE [2]. In our series, AE associated with NMDAR antibodies was noticed in 11/19 cases. One case showed an exceptional association of NF1 and anti-NMDAR encephalitis. To our best knowledge, this is the first case associating both conditions in the pediatric population. The pathogenetic significance of this association is not yet clarified. Herpes simplex virus encephalitis represents an important trigger for anti-NMDAR encephalitis. In our series, 2 cases had herpetic encephalitis prior to the onset of AE. Younger patients tend to present with seizures and abnormal movements, as seen in our cases, whereas adults typically present with psychiatric manifestations. Hyperkinetic movements, especially orofacial dyskinesias which consist of semirepetitive grimacing, chewing, or biting movements, are frequently noted in pediatric anti-NMDAR encephalitis [2]. In our series, orofacial dyskinesia was noted in 7/11 cases.

AE with anti-GAD65 antibodies is a rare condition in children, and to the best of our knowledge, only 16 pediatric cases were reported [11–15]. It is characterized by limbic involvement with refractory seizures, cognitive impairment, and behavioral disturbances [16]. All our cases of anti-GAD65 encephalitis presented with seizures. Extralimbic involvement like dysautonomia, as seen in one of our patients, was rarely reported [11].

Anti-Ma2 encephalitis was rarely reported in children with preferential involvement of limbic, diencephalic, and upper brainstem [2]. Our first case of anti-Ma2 encephalitis is the first reported Tunisian case and seems to be the youngest reported patient in the literature [17]. Pediatric cases presented with subacute onset of focal seizures, behavioral changes, speech disturbance, and dystonia [16]. All our cases presented with focal seizures. Two cases had behavioral changes, and one case had biopercular syndrome. Anti-Ma2 encephalitis is often associated with eye movement abnormalities, such as nystagmus, which were not observed in our cases [18].

Anti-SOX1 encephalitis is an exceptional condition in pediatric population. Only one pediatric case (17 years old) had been reported in literature [19]. In our series, we reported an exceptional case of anti-SOX1 encephalitis in a 12-year-old girl. It is the first reported Tunisian case and seems to be the youngest reported patient in the literature. Anti-SOX1 antibodies have been related to other neurological syndromes, including Lambert-Eaton myasthenic syndrome, polyneuropathy, and paraneoplastic cerebellar degeneration [20].

In AE, brain MRI typically includes T2 hyperintensity and rarely contrast enhancement [10]. It shows unilateral or asymmetrical increased signal of the temporal lobe on T2- and FLAIR-weighted images in 40% [3]. In our series, 9 cases had signal abnormalities in brain MRI.

In anti-NMDAR encephalitis, abnormalities of T2 signal involving cortical, subcortical, and infratentoriel regions and basal ganglia are found in about 30% of patients [2, 21]. Four anti-NMDAR encephalitis cases of our series had cortical and/or subcortical signal abnormalities. One patient had an increased T2 signal of the thalamus. In anti-Ma2 encephalitis, MRI abnormalities are frequent in the limbic system, diencephalon, and brainstem [16]. Brain MRI abnormalities were seen in 3/4 cases of anti-Ma2 encephalitis in our series. Some authors reported that brain positron emission tomography may be more sensitive than MRI in detecting abnormalities in AE [10].

CSF analysis in AE was often noncontributory, showing normal findings or increased protein level [5]. CSF white blood cell count and protein may be elevated, but they are typically less than 100/mm^3^ and 1 g/l, respectively [10]. In our series, hyperproteinorrhachia (maximum 0.84 g/l) was noted in 2 cases and pleocytosis (maximum 64/mm^3^) in 7 cases.

The EEG may be normal or show focal or generalized slowing in the temporal areas [9]. It is important for detecting subclinical seizures [10]. In our series, 15 patients had abnormal EEG. An EEG pattern known as extreme delta brush has been described in anti-NMDAR encephalitis. It represents a pattern of 1 to 3 Hz delta activity with superimposed 20 to 30 Hz *β* activity [10, 16]. This pattern had not been observed in our cases.

Because paraneoplastic encephalitis has been reported in children, screening for underlying tumor is necessary [9]. Anti-NMDAR antibody production can be stimulated by an underlying tumor, and the most frequently associated is ovarian teratoma [16]. For anti-Ma2 encephalitis, there is a strong association with testicular and germ cell tumors [18]. Patients with anti-GAD65 antibodies usually do not have underlying tumors [16]. Anti-SOX1 antibodies were highly associated with small cell lung cancer [10, 20]. In our series, oncological assessments were negative in all patients.

To date, there are no clear guidelines for the treatment of the autoimmune encephalitis. First-line immunotherapy includes a high dose of intravenous corticosteroids, intravenous immunoglobulin, or plasma exchange [2, 22]. Treatment should be initiated once a diagnosis of autoimmune encephalitis is suspected, as the result of autoantibody screening can take weeks [23]. In spite of appropriate first-line treatment, up to 35% of pediatric patients do not respond adequately. These patients often require more potent second-line therapies including rituximab and cyclophosphamide. Common protocols of rituximab include either weekly dosing for 4 weeks at 375 mg/m^2^ per dose or 750 mg/m^2^ for two doses 2 weeks apart, with a maximum of 1000 mg per dose in both protocols [4]. We used the first protocol of rituximab in 1 patient with anti-GAD65 encephalitis. Mycophenolate mofetil (MMF) and azathioprine may also be used, but there are inadequate data to make conclusions about their efficacy in AE [4, 24]. In our series, 7 cases received azathioprine.

There is growing evidence for novel agents including tocilizumab and bortezomib for the treatment of refractory AE [4].

The outcome of AE in childhood is generally good but may depend on the pathogenic autoantibody, neuronal target involved, and the time from symptom onset to treatment initiation [9]. Treatment of the underlying malignancy, if present, is crucial to achieve a better outcome [10]. The prognosis of anti-NMDAR encephalitis is often good in pediatric age, with 85% of full recovery. It was reported that CSF anti-NMDAR titers correlate strongly with the clinical disease course [16]. On the other hand, the clinical outcome is typically poor in patients with anti-Ma2 encephalitis, with medically refractory seizures [2]. In our series, 9 cases had a good outcome with full recovery.

The clinical utility of the following antibody titers is unclear [2]. An early decrease in antibody titers from CSF correlated with improved outcome in a study of patients with anti-NMDAR encephalitis. It may be useful to have titers at diagnosis and after recovery, as future elevation in titers may suggest a relapse, particularly in the setting of a recurrence of nonspecific symptoms [25].

## 5. Conclusion

Autoimmune encephalitis in children may present with a wide variety of symptoms. An early diagnosis and appropriate treatment of this disorder may prevent irreversible sequelae. Although the paraneoplastic origin is rare in childhood population, the presence of an underlying malignancy must be ruled out.

## Figures and Tables

**Table 1:** Demographic, clinical, and laboratory features, imaging and accessory tests, treatment, and outcome of 19 children with autoimmune encephalitis.

Case	Gender	Age at diagnosis	Antibodies	Intensity of positive test	Seizure	Associated features	CSF findings	MRI findings	Treatment	Outcome/follow-up duration
1	F	10 months	Anti-NMDAR	CSF: ++	Yes	Behavioral disorders, speech disorders, orofacial dyskinesia, dystonia chorea	Normal	Increased temporal T2 signal	Methylprednisolone IVIG	Full recovery/3 years
2	M	9 years	Anti-NMDAR	CSF: +++ Serum: +	Yes	Behavioral disorders, decreased level of consciousness, sleepiness, speech disorders, orofacial dyskinesia, dystonia	Normal	Normal	Methylprednisolone IVIG	Partial recovery/3 months
3	M	8 years	Anti-NMDAR	CSF: +++	Yes	Behavioral disorders, decreased level of consciousness, orofacial dyskinesia	Hyperproteinorrhachia, pleocytosis	Normal	Methylprednisolone IVIG	Full recovery/6 years
4	M	12 years	Anti-NMDAR	CSF: ++ Serum: ++	Yes	Behavioral disorders, hallucination, speech disorders, dystonia	Normal	Normal	Methylprednisolone IVIG	Full recovery/3 years
5	F	13 years	Anti-NMDAR	CSF: + Serum: -	Yes	Behavioral disorders, decreased level of consciousness	Normal	Normal	Methylprednisolone IVIG Azathioprine	Full recovery/4.5 years
6	F	5.5 years	Anti-NMDAR	CSF: + Serum: -	Yes	Behavioral disorders, speech disorders, sleepiness	Normal	T2/FLAIR hyperintensity with no contrast enhancement in deep white matter	Methylprednisolone IVIG Azathioprine	Partial recovery/6.5 years
7	F	13 years	Anti-NMDAR	CSF: + Serum: -	Yes	Behavioral disorders, orofacial dyskinesia	Pleocytosis	Normal	Methylprednisolone IVIG	Full recovery/6 years
8	M	8.5 years	Anti-NMDAR	CSF: + Serum: -	Yes	Behavioral disorders, speech disorders, orofacial dyskinesia, sleepiness	Pleocytosis	Normal	Methylprednisolone IVIG	Full recovery/3 months
9	F	13 years	Anti-NMDAR	CSF: +	No	Behavioral disorders, speech disorders, sleepiness	Normal	Increased temporal T2 signal	Methylprednisolone IVIG	Partial recovery/9.5 years
10	F	6 years	Anti-NMDAR	CSF: + Serum: +/-	Yes	Behavioral disorders, decreased level of consciousness, speech disorders, dystonia, orofacial dyskinesia	Hyperproteinorrhachia	Increased temporal T2 signal	Methylprednisolone IVIG Azathioprine	Full recovery/4.5 years
11	F	8 years	Anti-NMDAR	CSF: +++ Serum: -	Yes	Behavioral disorders, hallucination, decreased level of consciousness sleepiness, speech disorders, orofacial dyskinesia	Pleocytosis	Increased thalamus T2 signal	Methylprednisolone IVIG	Full recovery/3 months
12	F	6 years	Anti-Ma2	Serum: +	Yes	Behavioral disorder, speech disorders	Normal	Normal	Methylprednisolone IVIG	Partial recovery/5.5 years
13	M	8 years	Anti-Ma2	Serum: ++	Yes	Behavioral disorders, decreased level of consciousness, hallucination	Pleocytosis	External capsule Increased signal	Methylprednisolone IVIG Azathioprine	Full recovery/1.5 years
14	F	23 months	Anti-Ma2	CSF: + Serum: +	Yes	Decreased level of consciousness, speech disorders, behavioral disorders, hallucination	Normal	Increased left temporoparietal, basifrontal, and occipital T2 signal with leptomeningeal enhancement	Methylprednisolone	Partial recovery/3 months
15	M	2.5 years	Anti-Ma2	CSF: ++ Serum: +	Yes	Behavioral disorders, decreased level of consciousness, speech disorders, swallowing and chewing disorders, drooling	Normal	Increased right frontal signal	Methylprednisolone IVIG Azathioprine	Partial recovery/6.5 years
16	F	4.5 years	Anti-GAD65	CSF: 45 UI/ml (NV < 10 UI/ml)	Yes	Behavioral disorders, decreased level of consciousness, orofacial dyskinesia, sleepiness	Normal	Normal	Methylprednisolone IVIG Azathioprine	Partial recovery/6 years
17	F	9 years	Anti-GAD65	CSF: 32 UI/ml (NV < 10 UI/ml)	Yes	Behavioral disorders, decreased level of consciousness, speech disorders, autonomic disturbances, hallucination, sleepiness	Normal	Normal	Methylprednisolone IVIG Rituximab	Deceased/6 months
18	M	9.5 years	Anti-GAD65	CSF: 71 U/ml (NV < 10 UI/ml)	Yes	Decreased level of consciousness, acute confusional state, visual hallucinations, behavioral disorders, tremor	Pleocytosis	Normal	Methylprednisolone IVIG Azathioprine	Partial recovery/1.5 years
19	F	12 years	Anti-SOX1	Serum: +++	Yes	Behavioral disorders, decreased level of consciousness, distal upper limb tremor and myoclonia	Pleocytosis	Increased temporal T2 signal	Methylprednisolone IVIG	Partial recovery/1 year

M: male; F: female; CSF: cerebrospinal fluid; MRI: magnetic resonance imaging; FLAIR: fluid-attenuated inversion recovery; NV: normal value; NMDAR: N-methyl-D-aspartate receptor; GAD65: glutamic acid decarboxylase 65; IVIG: intravenous immunoglobulin; SOX1: Sry-like high mobility group box 1.

## Data Availability

The data supporting the findings of this study are available within the article.
